# Can pain self-efficacy mediate the influence of pain intensity on outcomes for patients with musculoskeletal pain? A prospective mediation analysis with 12 months follow-up

**DOI:** 10.1007/s11136-025-04118-3

**Published:** 2026-01-03

**Authors:** Monica Unsgaard-Tøndel, Kari Anne I. Evensen, Nina Køpke Vøllestad, Hilde Stendal Robinson, Fredrik Granviken, Astrid Woodhouse, Ingebrigt Meisingset

**Affiliations:** 1 Department of Neuromedicine and Movement Science, Faculty of Medicine and Health Sciences, Norwegian University of Science and Technology, NTNU, Trondheim, Norway; 2 Department of Public Health and Nursing, Faculty of Medicine and Health Sciences, Norwegian University of Science and Technology, NTNU, 7491 Trondheim, Norway; 3 Department of Physiotherapy, Trondheim Municipality, Trondheim, Norway; 4 Department of Clinical and Molecular Medicine, NTNU, Trondheim, Norway; 5 Children’s Clinic, St. Olavs Hospital, Trondheim University Hospital, Trondheim, Norway; 6 Department of Rehabilitation Science and Health Technology, Oslo Metropolitan University, Oslo, Norway; 7 Department of Public Health Science and Interdisciplinary Health Science, Institute of Health and Society, University of Oslo, Oslo, Norway; 8 Clinic of Rehabilitation, St Olav’s Hospital, Trondheim University Hospital, Trondheim, Norway; 9 Department of Circulation and Medical Imaging, Faculty of Medicine and Health Sciences, Norwegian University of Science and Technology, NTNU, Trondheim, Norway

**Keywords:** Pain self-efficacy, Health-related quality of life, Patient-specific function, Global perceived effect, Musculoskeletal pain, Mediation analysis

## Abstract

**Purpose:**

In longstanding pain, a core treatment aim is to minimize the effect of pain on health-related quality of life and patient-specific function. The study aim was to assess whether the effect of pain intensity on outcome is mediated through pain self-efficacy. A secondary aim was to assess the mediating effect of pain self-efficacy for a subgroup of patients with low pain self-efficacy.

**Methods:**

Prospective data of 422 patients with musculoskeletal pain seeking physical therapy was applied in analyses. The exposure was baseline pain intensity and outcomes were health-related quality of life (EQ-5D), patient-specific function (PSFS) and global perceived effect (GPE) at 12 months. The mediator was measured by the 2-item pain self-efficacy questionnaire (PSEQ-2) at baseline and 3 months.

**Results:**

The mediating effect of pain self-efficacy on the relation between pain intensity and health-related quality of life and patient-specific function was statistically, but not clinically, significant: − 0.01 (95% CI − 0.01 to − 0.00) and − 0.08 (95% CI (− 0.15 to − 0.01). For the mediating effect of pain self-efficacy on the relation between pain intensity and global perceived effect, odds ratio was 0.97 (95% CI 0.92 to 1.01). In a subgroup with low baseline pain self-efficacy, the mediating effect of pain self-efficacy was − 0.28 (− 0.55 to − 0.01) for patient-specific function.

**Conclusion:**

Pain self-efficacy did not have an important mediating effect on the relation between pain intensity and outcome. In a subgroup with reduced pain self-efficacy, the effect of baseline pain intensity on patient-specific function was mediated by pain self-efficacy.

**Trial registration:**

ClinicalTrials.gov Identifier: NCT03626389. Registered August 13, 2018 (retrospectively registered).

**Supplementary Information:**

The online version contains supplementary material available at 10.1007/s11136-025-04118-3.

## Background

Longstanding musculoskeletal pain is a global public health problem with substantial negative consequences for the individuals’ physical function and health-related quality of life [1]. In Norway, musculoskeletal pain is the third most expensive condition for inhabitants below the age of 70 years [2]. As the prevalence of musculoskeletal disorders is continuously increasing, primary health care services for these patients need upscaling [1]. Long-term improvement in health-related quality of life and physical function is a core aim for treatment [3]. Evaluation of treatment effect should combine individualized outcome measures like the patient-specific functional scale [4] with generic outcomes for health-related quality of life [5] and global perceived effect [6].

The relation between pain intensity and health-related quality of life is complex [7] and influenced by psychological factors. Psychological factors have been reported to predict health-related quality of life [8] and to mediate the effect of pain on health-related quality of life in fibromyalgia [9] and various medical conditions including diabetes, cardiovascular diseases, cerebrovascular diseases, pulmonary conditions, chronic pain, and cancer [10]. In line with this, it has been suggested that pain-related beliefs are more important determinants of reduced physical function than pain intensity in musculoskeletal pain [11]. Screening for psychosocial factors is recommended to inform clinical decision-making and tailor management [12]. Consequently, the modifiable mechanisms behind cognitive factors’ influence on outcome should be explored and used to inform management strategies and optimize health-related quality of life and physical function for persons with- or at risk for chronic pain. Pain self-efficacy is a potentially modifiable factor and has previously been associated with prognosis and outcome for musculoskeletal pain [13, 14]. The term self-efficacy stems from social cognitive learning theory and was introduced as a theory of behavioral change [15]. It explains how motivation, effort and endurance are influenced by the confidence that individuals hold towards their own competence. Some previous studies have indicated that health-related quality of life and functional outcome can be mediated by self-efficacy: An exploratory analysis of a randomized controlled trial found that pain self-efficacy mediated the effect of the intervention on health-related quality of life for patients with chronic headache [16] and a cross-sectional study of adolescents with persistent pain suggested that self-efficacy mediated the associations between pain intensity and health-related quality of life [17]. However, a cross-sectional design is not suited to exploring mediating effects [18].

Self-efficacy can mediate the effect of pain on low back pain-related disability, as well as the effects of various interventions, education, and graded sensorimotor training on pain and disability for back pain [19–21]. In addition, mediating effects for self-efficacy have been observed for cognitive-behavioral based physical therapy on physical health after spine surgery [22]. These previous studies explored mediating effects in various groups of patients seeking treatment for low back- and neck pain in different settings [19–22]. However, whether, and for whom, pain self-efficacy has an overall mediating influence on the effect of musculoskeletal pain on long-term outcome is unknown. The timing of the mediator assessment is important [22], and prospective studies with intermediate registrations of mediators and long-term follow-up are needed to explore possible causal pathways for successful outcome [23]. To provide effective and person-centered management for patients seeking physical therapy for musculoskeletal pain, we need insight into the contribution from modifiable mediators for sustained, successful, and patient-specific outcomes. Meaningful limits for low level of pain self-efficacy have been proposed [24], and whether exceeding this limit has clinical importance in this patient group is not known. Acknowledging this knowledge gap, the overall purpose of this study was to assess whether pain self-efficacy has mediating effect across different musculoskeletal pain conditions and on generic as well as individualized outcomes in the long term.

On this background, we aimed to investigate if pain self-efficacy could mediate the effect of pain intensity on health-related quality of life, patient-specific function and global perceived effect after 12 months for patients seeking physical therapy for musculoskeletal pain. A secondary aim was to assess if pain self-efficacy mediated the effect of pain intensity on outcome in a subgroup of patients with low pain self-efficacy at baseline.

## Methods

### Study design

The present study is a secondary mediation analysis from a prospective longitudinal observational cohort study, the Research program for Physiotherapy in Primary Health Care (FYSIOPRIM) [25]. FYSIOPRIM collected data from patients receiving physical therapy in outpatient clinics in primary health care in the period 2015–2020. The physical therapist provided project information and consent forms to their patients on first consultation. Participants received questionnaires at baseline, 3, 6 and 12-month follow-ups. In the present study, data at baseline, 3 and 12 months were utilized. The temporal order with baseline registration of exposure, intermediate registration of mediator at 3 months, and 12 months follow-up registrations of all outcomes was applied to enhance the causal interpretability of the mediation analysis.

### Participants

A total of 4071 patients seeking physical therapy and included in FYSIOPRIM [25] were considered for inclusion in the study, based on the following inclusion criteria: Seeking physical therapy for musculoskeletal symptoms and provided responses to study variables at all timepoints, specifically: pain intensity at baseline, self-efficacy at 3 months, and outcome data at 12 months. Musculoskeletal symptoms were defined as having an ICPC-2 diagnosis from the L-category. A total of 2748 individuals had musculoskeletal pain as their primary cause for seeking treatment. To be eligible for our study, patients needed data on baseline pain intensity and all relevant confounders assessed at baseline (*n* = 1062). In addition, patients needed data on self-efficacy at 3 months and outcome data at 12 months (*n* = 422). The Pain Self-Efficacy Scale was introduced after the study began; therefore, the first participants enrolled in Fysioprim did not report pain self-efficacy, resulting in data missing completely at random. In total 422 patients were included in the final sample for analysis. We have described the baseline characteristics for the 422 patients compared to the characteristics of the total sample with MSK pain and the sample with all baseline information but missing data at the follow-up assessments (see Online resource E).

### Procedures/data collection

Questionnaires were completed by patients either by using an e-tablet or a web-link sent by e-mail [25]. The data were collected at three timepoints: At baseline, 3 months, and 12 months after inclusion.

**Fig. 1:**
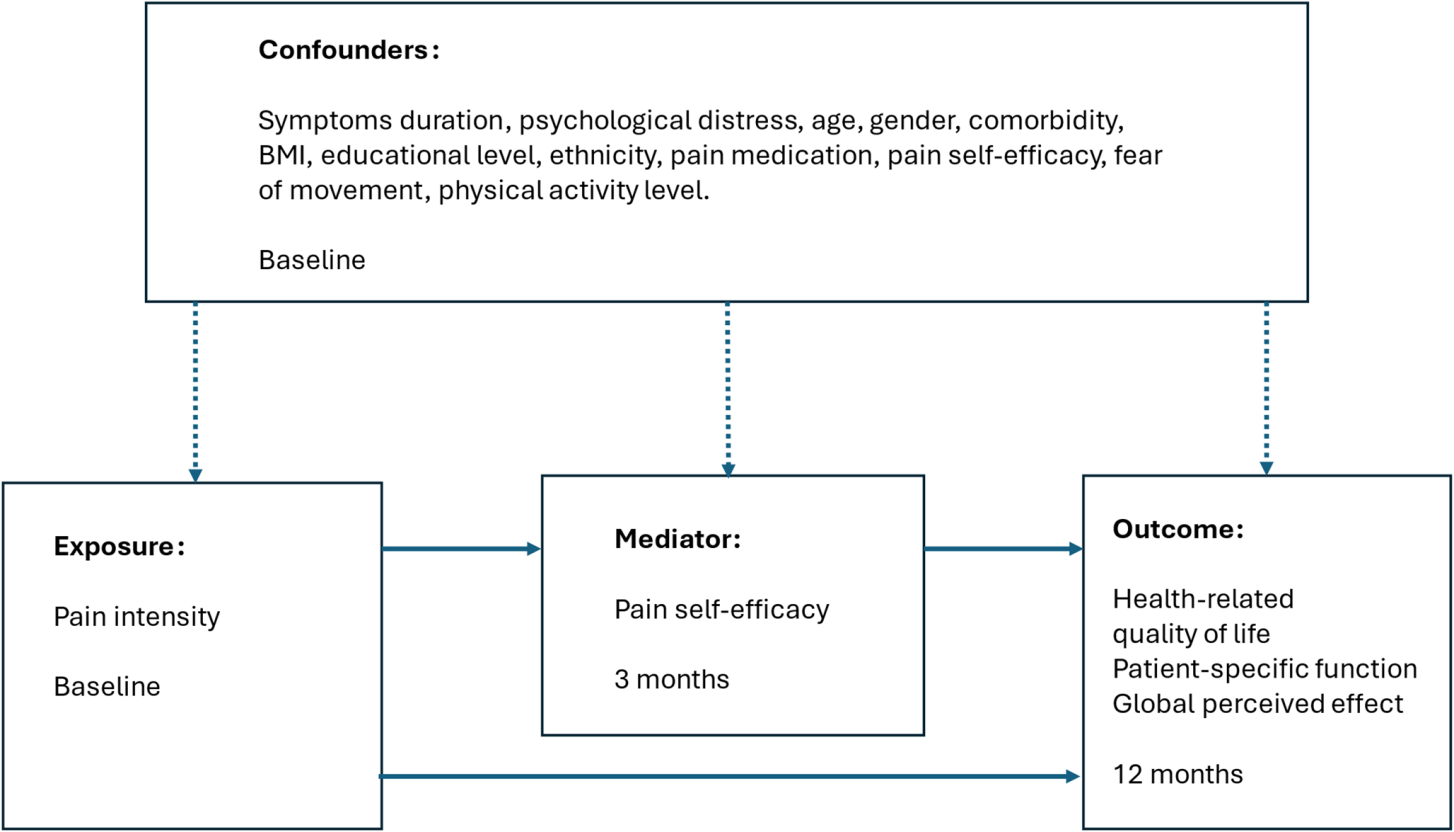
Directed acyclic graph of indirect (mid solid arrows) and direct effect (lower solid arrow) of baseline pain intensity on 12 months outcome. Vertical dashed arrows represent the influence of confounders on exposure, mediator, and outcome. For the mediation analysis, the two mid solid arrows represent the influence of baseline pain intensity on pain self-efficacy at 3 months (left solid arrow) and the influence of pain self-efficacy at 3 months on outcome at 12 months (right solid arrow). Together, these arrows represent the indirect effect, i.e. the mediating effect of pain self-efficacy on the relation between baseline pain intensity and outcome at 12 months

### Exposure, mediator, and outcomes

Table 1 gives an overview of the variables and measurements in the study.

*Baseline pain intensity* Pain intensity was assessed at start of treatment with the question “How much pain have you had the last week” and measured by the Numeric pain Rating Scale (NRS) on an 11-point numeric rating scale ranging from 0 to 10, where 0 is no pain and 10 is worst possible pain [26].

*Pain self-efficacy* Pain self-efficacy was measured with the 2-item version of the pain self-efficacy questionnaire (PSEQ-2) which includes the following statements [24]: “*I can do some form of work*,* despite pain”* (work includes housework and paid and unpaid work), and “*I can live a normal lifestyle*,* despite pain”*. Responses ranged from 0 to 6 were 0 represented *“not at all confident”* and 6 represented *“completely confident”.* The total score thus ranges from 0 to 12 and higher score indicates higher level of self-efficacy. We defined a PSEQ-2 score of 8 or more as high pain self-efficacy [24]. Pain self-efficacy was defined as mediator a priori and measured at baseline and after 3 months. The baseline measurement was utilized to define a subgroup with low pain self-efficacy at baseline, while the 3 months measurement was utilized as mediator in analyses.

*Health-related quality of life* was measured by the EQ5D-5L instrument [27]. The score from the five dimensions was transformed to an index score (range: − 0.285 to 1.00) using the English population value set [27], and a score of 1.00 indicates *”full health”*. The mean minimal important difference for the EQ-5D has been suggested to be 0.08 [28]. There was no Norwegian value set available when analysis was performed, therefore we used the UK value set.

*Patient-specific function* was assessed by the patient-specific functional scale (PSFS) [4, 29]. The physical therapist asked the patient to prioritize three activities that were limited by the problems they were seeking physical therapy for. The degree of reduced function is ranged on an 11-point numeric point rating scale [29]. 0 = Can not perform activity. 10 = Can perform without difficulty. We used the first activity with scores in the present study.

*Global perceived effect* Patients were asked to score their improvement after 12 months on a 1 to 7 scale, where 1 represented “very much better” and 7 represented “very much worse” [6]. This variable was dichotomized into the following categories: Perceived treatment effect (very much better or much better) and not perceived treatment effect (slightly better to very much worse).

**Table 1:** Exposure, mediators, outcome, confounders and timing of assessment

Variable domain	Measurement instrument	Timing of assessment^a^	Measurement level applied in analysis
*Exposure*			
Pain intensity	Numeric pain Rating Scale (NRS) Range 0–10; 0 = no pain and 10 = worst imaginable pain	T0	Continuous
*Mediator*			
Pain self-efficacy	Pain Self-Efficacy Questionnaire 2 item version (PSEQ-2): 1. I can do some form of work, despite pain (work includes housework and paid and unpaid work) 2. I can live a normal lifestyle, despite pain Range: 0–6; 0 = not at all confident, 6 = completely confident	T1	Continuous
*Outcome*			
Health-related quality of life	EuroQol EQ5D 5 L - Health related quality of life; 5 items of mobility, self- care, usual activities, pain and anxiety/depression. Each item has 5 response categories Summarized as index score: − 0.285 to 1.00; 0 = death and 1 = full health (negative scores = worse than death)	T2	Continuous
Patient-Specific Function	Patient-specific functional scale (PSFS). Patient-listed activity limitations scored on a 0–10 scale.	T2	Continuous
Global Perceived Effect	1 = very much better, 2 = much better, 3 = slightly better, 4 = neither better nor worse, 5 = slightly worse, 6 = much worse. 7 = very much worse. Dichotomized: perceived treatment effect (1–2) or not (3–7).	T2	Binary
*Confounders*			
Symptom duration	Item 1 from Örebro Screening Questionnaire: How long have you had your current pain problem? 0–4 weeks, 4–11 weeks, 3–6 months, 6–12 months, over 1 year.	T0	Categorical
Age	Age in years	T0	Continuous
Gender	Female Male	T0	Binary
Psychological distress	Hopkins symptom checklist, 10 item version [30]. Range: 1.0–4.0; 1 = not at all and 4 = very much (extremely). Mean item score is calculated	T0	Continuous
BMI		T0	Continuous
Educational level	Dichotomous: Defined as higher education or not	T0	Binary
Pain medication	Last week, yes or no.	T0	Binary
Fear of movement	1 question [31]: How much fear do you have that these complaints would be increased by physical activity? NRS: 0–10; 0 = no fear and 10 = very much fear	T0	Continuous
Physical activity level	How often are you physically active? (1) Never. (2) Less than once per week. (3) Once per week. (4) 2–3 times per week. (5) Approximately each day. Defined as physically active (4–5) or not (1–3).	T0	Binary
*Additional confounders added in sensitivity analyses to estimate the magnitude of unmeasured confounding*			
Sleep	Sleep item from 15D [32]. Participants were asked to describe their sleep with five response options in a Likert scale were 1 represents “I am able to sleep normally, e.g., I have no problems with sleeping” and 5 represents “I suffer severe sleeplessness, e.g., sleep is almost impossible even with full use of sleeping pills, or staying awake most of the night”. Dichotomised: No sleep problems (score 1–2), or moderate to severe sleep problems (3–5).	T0	Binary
Number of pain sites	Mark the relevant body region(s) for your complaint (multiple answers possible). Head, neck, shoulder/upper arm, upper back, elbow/underarm, wrist/hands, hip/thigh, low back, ankle /feet, knee Categories: 1) 1, 2) 2–3, 3) 4–5, 4) 6–10	T0	Categorical

T0 = Baseline, T1 = 3 months, T2 = 12 months

### Confounders

The potential confounders were selected based on consideration of plausible mechanisms of effect and selection of variables that could potentially influence at least two out of three of the exposure, mediator and outcome [33]. Potential confounders were selected based on the preparation of the Directed Acyclic Graph (DAG, Fig. 1) and Table 1, and cover the following domains: Demographics, pain characteristics, pain–related cognitions, psychological factors, social factors, and lifestyle factors registered at baseline. Literature supporting the selection of confounders is referred to in Online resource D.

### Statistical analysis

The mediation analysis was based on a counterfactual-causal framework [34], which is the recommended and state-of-the-art mediation analysis [35]. In the counterfactual approach, the estimated mediation effect is separated from the potential interaction effect by four-way decomposition. In four-way decomposition, the total effect of the exposure on the outcome is specified into components due to mediation alone, to interaction alone, to both mediation and interaction, and to neither mediation nor interaction (direct effect) [34]. The results from the analyses are presented as beta coefficient (health-related quality of life and patient-specific function) or odds ratio (global perceived effect) for change in outcome that are explained by the mediator alone after adjusted for the other effects (interaction and both mediation and interaction).

To enable a true causal interpretation of the mediation analysis, there are several assumptions that must be fulfilled [34]:

- Temporal precedence between exposure, mediator and outcome to avoid reverse causation [18, 33]. Our analysis accommodates this by using 3 consecutive timepoints as outlined in the DAG (Fig. 1).
- The effect of the exposure on the outcome has no unmeasured confounding.
- The effect of the exposure on the mediator has no unmeasured confounding.
- The effect of the mediator on the outcome has no unmeasured confounding.
- None of the mediator-outcome confounders are themselves affected by the exposure [34].

We have accommodated the assumptions by adjusting for potential confounders assessed at baseline. Our data consists of a comprehensive set of biopsychosocial patient characteristics that enable us to adjust for potential confounders for all types of confounding. We used DAGs to identify potential confounders based on literature search, expert knowledge, and discussions with researchers and clinicians. Our analyses are not automatically protected against residual confounding. We therefore conducted sensitivity analyses to estimate whether residual confounding is likely to change the main results. When constructing the DAGS we discussed the risk of the exposure pain intensity influencing the confounders. Therefore, we chose not to include sleep and number of pains sites in the main analyses.

We present two linear regression models for each of the analyses with health-related quality of life and patient specific function as outcome (Table 4, columns 1 and 2, rows 1–4 and Table 5, rows 1–2), and logistic regression models for global perceived effect outcome (Table 4, columns 1 and 2, rows 5–6 and Table 5, row 3): One model assessed the effect of the exposure on the mediator by modeling the mediator as a function of the exposure and confounders (the mediator model). The other model assessed the effect of the mediator on the outcome, by modeling the outcome as a function of the exposure, confounders, and the mediator (the outcome model).

We estimated the size of the change in pain self-efficacy from baseline to 3 months needed to produce a clinically significant improvement in outcome at 12 months. For the health-related quality of life outcome, this was estimated by calculating the magnitude of change in self-efficacy needed to produce an improvement on EQ5D to be considered clinically important, i.e. a change of at least 0.08 points [28, 36]. This value was divided by the mediator coefficient from the regression model for the mediator (Table 4). We also calculated the change in PSEQ needed to obtain clinically significant change in PSFS by dividing the predefined values for minimal clinically significant change on the mediator coefficient from the outcome regression models [37].

For the decomposition analyses we estimated the causal effects for a change in pain intensity from 4 to 6 on the NRS, as 2-point difference in pain intensity is reported to be a minimal clinically important difference [38]. In supplementary analyses we also explored the mediation effect when applying the possible range of exposure values for pain intensity on a scale from 0 to 10 and a 2-point difference. Confounders were set at their mean value for continuous variables and as a reference value for categorical variables. The mediator self-efficacy was set at the mean value. The included sample with complete data was compared to the whole sample with musculoskeletal pain (online resource E).

Considering the relatively high mean score on PSEQ-2, we performed a subgroup analysis to investigate the mediation effect for patients with low self-efficacy at baseline. Low self-efficacy was defined as a score below 8 [24].

### Sensitivity analysis

The influence of unmeasured confounders was estimated by adding two possible confounders in the model, one by one: Sleep [39, 40], and number of pain sites [41] (Table 4). Due to non-normally distributed mediator and outcome variables, we performed sensitivity analyses with mediator and continuous outcomes dichotomized, independently and in separate models (Online resource A and B). We also compared baseline characteristics between patients with missing data at follow-up and patients with complete data (Online resource E).

The analyses were performed in STATA version 17 with the Med4way command [35]. *P*-values < 0.05 were considered statistically significant.

## Results

A total of 422 participants with complete data on study variables were included in the study (Table 2). Eligible patients with missing data on follow-up had no significant differences for baseline characteristics compared to those with complete follow-up data (Online resource E). The participants’ mean age was 48.6 years (SD 16.6), 71.6% were female, and 23.9% reported to be physically inactive at baseline. The scores on exposure, mediator and outcome variables are presented in Table 3 for all 3 measurement points. Pain intensity was 4.3 (SD 2.2) at baseline, and 3.0 (SD 2.6) at 12-month follow-up. Pain self-efficacy was 9.5 (SD 2.6) at baseline and 10.4 (SD 2.5) at 12 months follow-up.

**Table 2:** Baseline characteristics of participants

	Total sample (*N* = 422)
Age, mean (SD)	48.6 (16.6)
Female gender, n (%)	302 (71.6)
Body mass index, mean (SD)	26.2 (4.7)
Higher education, n (%)	259 (61.4)
Physically inactive, n (%)^a^	101 (23.9)
Fear of movement, mean (SD)^b^ (0–10)	3.1 (2.8)
Use of pain medication last week, n (%)	190 (45.0)
Number of pain sites (0–10), mean (SD)	3.0 (2.3)
*Pain duration, n (%)*	
< 3 months	34 (8.1)
3–12 months	180 (42.7)
> 12 months	208 (49.3)
Mental distress, HSCL (1.0–4.0)^c^, mean (SD)	1.65 (0.54)
Sleep problems, n (%) ^d^	150 5.6)

^a^Physically active once per week or less

^b^1 question: How much fear do you have that these complaints would be increased by physical activity? NRS: 0–10; 0 = no fear and 10 = very much fear

^c^Hopkins symptom checklist, 10-item version. Range: 1.0–4.0; 1 = not at all and 4 = very much (extremely), mean item score is calculated

^d^Sleep item from 15D [32]. Participants were asked to describe their sleep with five response options in a Likert scale were 1 represents “I am able to sleep normally, e.g., I have no problems with sleeping” and 5 represents “I suffer severe sleeplessness, e.g., sleep is almost impossible even with full use of sleeping pills or staying awake most of the night”. Dichotomized: No sleep problems (score 1–2), or moderate to severe sleep problems (3–5)

**Table 3:** Mean values (SD) of the exposure, mediator and outcomes at baseline and follow-ups

Total sample (*n* = 422)	Baseline	3 months	12 months
Pain intensity (0–10)^a^	4.3 (2.2)	3.4 (2.5)	3.0 (2.6)
Pain self-efficacy (0–12)^b^	9.5 (2.6)	10.0 (2.7)	10.4 (2.5)
Health related quality of life, (− 0.285–1.00)^c^	0.65 (0.18)	0.77 (0.18)	0.76 (0.19)
Patient Specific Functional Scale, range (0–10)^d^	3.7 (2.4)	7.0 (2.5)	7.3 (2.8)
Global perceived effect, perceived treatment effect (n, %)^e^	Not applicable	263 (62.6)	285 (68.5)

^a^Measured by NPRS = Numeric pain rating scale: 0 = no pain, 10 = worst imaginable pain

^b^Measured by PSEQ-2 = 2-item short form Pain self-efficacy Questionnaire. 12 = best possible score

^c^Measured by EQ5D = EuroQoL5D: 1 = best possible score

^d^Patient-specific functional scale, PSFS. 10 = best possible score

^e^Global perceived effect, GPE, dichotomized

The mediation effect of self-efficacy on the relation between baseline pain intensity and health-related quality of life at 12 months showed a significant, but small effect, β = − 0.01 (− 0.01 to 0.00), Table 4. The sensitivity analyses including sleep and number of pain sites as confounders influenced the results marginally (Table 4). The sensitivity analyses with dichotomized mediator and outcome showed marginally weaker relations (Online resource A and B).

**Fig. 2:**
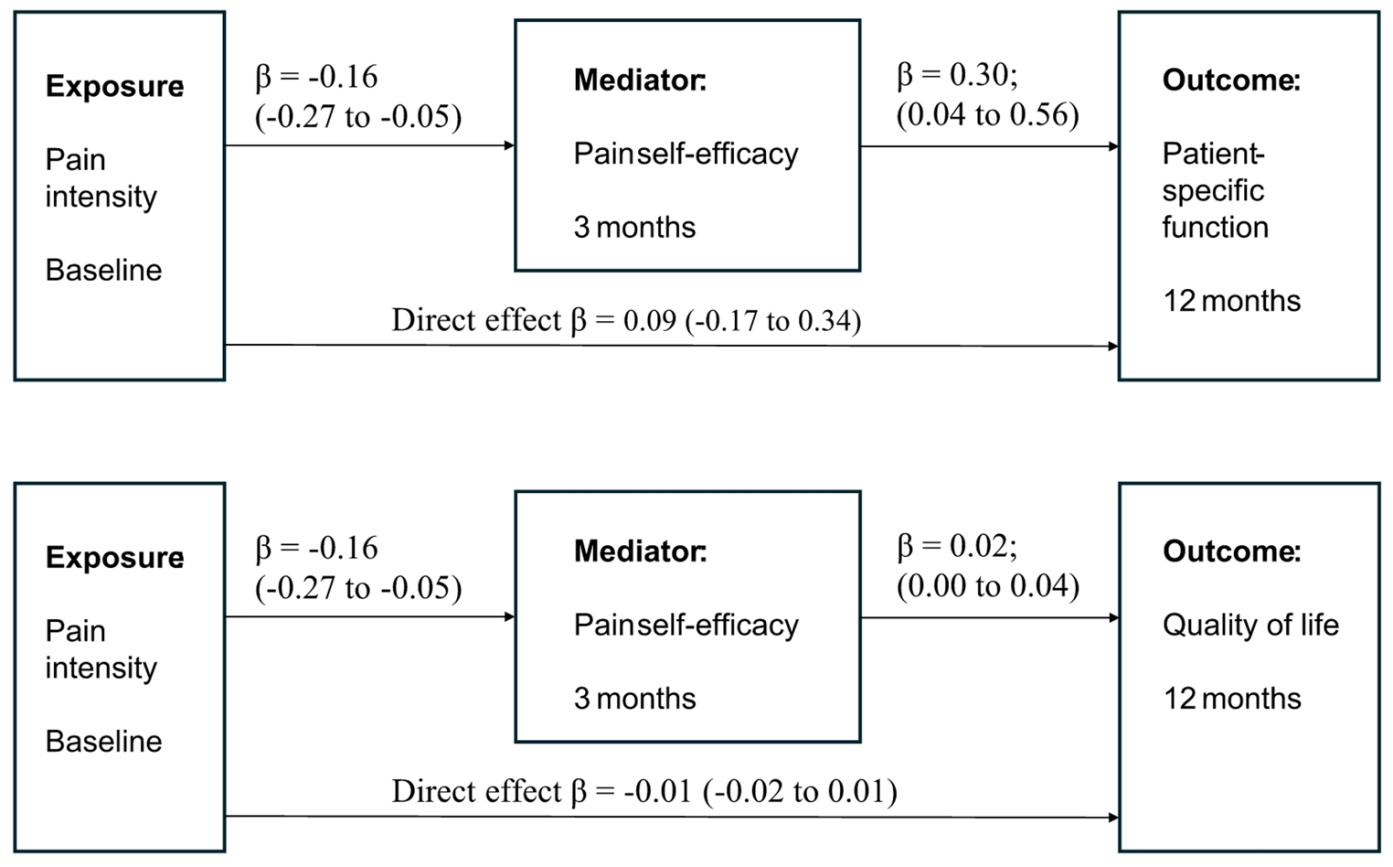
Effect estimates with 95% confidence intervals of indirect (upper arrows) and direct (lower arrow) effect of baseline pain intensity on 12 months health-related quality of life and patient-specific function outcomes. The two upper arrows represent the mediating effects in two steps: the influence of baseline pain intensity on pain self-efficacy at 3 months (left arrow) and the influence of pain self-efficacy at 3 months on outcome at 12 months (right arrow). Indirect effects were adjusted for confounding and interaction. Direct effects were adjusted for mediation, interaction, and confounding

The mediation effect of self-efficacy on the relation between baseline pain intensity and patient-specific function at 12 months also showed a statistically significant, but small effect (β = − 0.08; 95% CI = − 0.15 to − 0.01) (Table 4). Including sleep and number of pain sites as additional confounders (Table 4) or dichotomizing mediator or outcomes (Online resource A and B) did not influence the results substantially for the patient-specific functional outcome (Fig. 2).

The mediation effect of self-efficacy on the relation between baseline pain intensity and global perceived effect at 12 months showed no effect (Odds ratio = 0.97; 95% CI = 0.92 to 1.01).

Supplementary analyses exploring the possible range of exposure values for pain intensity on a scale from 0 to 10 and a 2-point difference yielded similar effect estimates (results not shown). The estimates for mediated interaction and reference interaction in the four-way decomposition also showed no significant effects (Table 4, online resource F). The baseline values for final sample were comparable to the values for the whole sample (online resource E).

**Table 4:** Effect decomposition in whole sample: mean difference on outcome health-related quality of life, patient-specific function and global perceived effect. Sensitivity analyses with the confounders sleep and number of pain sites

Mediating effect of pain self-efficacy at 3 months on outcome	Exposure^a^- (pain intensity)-mediator** effect	Mediator^b^ -outcome effect	Average total effect	Average direct effect	Pure indirect effect
Health-related quality of life^c^ (*n* = 422)	− 0.16 (− 0.27 to − 0.05)	0.02 (0.00 to 0.04)	− 0.013 (− 0.03 to 0.00)	− 0.01 (− 0.02 to 0.01)	− 0.01 (− 0.01 to 0.00)
Health-related quality of life (*n* = 404) Sensitivity	− 0.15 (− 0.26 to − 0.03)	0.017 (0.00 to 0.03)	− 0.010 (− 0.03 to 0.01)	− 0.01 (− 0.02 to 0.01)	− 0.01 (− 0.01 to 0.00)
Patient-specific function^d^ (*n* = 418) (95% CI)	− 0.16 (− 0.27 to − 0.05)	0.30 (0.04 to 0.56)	− 0.01 (− 0.27 to 0.25)	0.09 (− 0.17 to 0.34)	− 0.08 (− 0.15 to − 0.01)
Patient-specific function (*n* = 400) (95% CI) Sensitivity	− 0.16 (− 0.27 to − 0.05)	0.31 (0.04 to 0.58)	− 0.04 (− 0.31 to 0.24)	0.07 (− 0.20 to 0.33)	− 0.08 (− 0.15 to − 0.01)
Global perceived effect^e^ (*n* = 416), Odds ratio	β = − 0.15 (− 0.26 to − 0.04)	1.12 (0.88 to 1.44)	0.84 (0.68 to 1.05)	0.89 (0.73 to 1.08)	0.97 (0.92 to 1.01)
Global perceived effect^3^ (*n* = 398), Odds ratio Sensitivity	β = − 0.15 (− 0.26 to − 0.04)	1.11 (0.86 to 1.44	0.84 (0.67 to 1.06)	0.88 (0.72 to 1.07)	0.98 to 1.02)

^a^Exposure: Pain intensity, Numeric pain rating scale, range 0–10. Lower score = better

^b^Mediator: Pain self-efficacy, 2-item pain self-efficacy questionnaire, 0–12. Higher score = better

^c^Health-related quality of life, EQ5D, range − 0.28 to 1.00). Higher score = better., β-estimate

^d^Patient-specific functional scale, PSFS, range 0–10. Higher score = better. β-estimate

^e^Global perceived effect, GPE, dichotomized: perceived treatment effect or not. OR = odds ratio

### Subgroup analysis of patients with low level of pain self-efficacy at baseline

Table 5 shows the subgroup analysis where we estimated the causal effect for patients with low self-efficacy at baseline (PSEQ score < 8). The overall results for health-related quality of life as outcome did not differ from the whole sample, as the decomposition analyses revealed no significant results. For PSFS, the analysis showed a mediation effect of self-efficacy on the relation between pain intensity at baseline and patient-specific function at follow-up, β − 0.28 (− 0.55 to − 0.01).

**Table 5:** Effect decomposition: mean difference on outcome* health-related quality of life, patient-specific function and global perceived effect in subsample with pain self-efficacy less than 8 at baseline

Mediating effect on pain self-efficacy at 3 months on outcome	Exposure (pain intensity)-mediator effect	Mediator-outcome effect	Average total effect	Average direct effect	Pure indirect effect
Health-related quality of life^a^ (*n* = 90)	− 0.45 (− 0.76 to − 0.14)	0.02 (− 0.01 to 0.05)	− 0.03 (− 0.08 to 0.02)	− 0.01 (− 0.05 to 0.03)	− 0.02 (− 0.05 to − 0.003)
Patient-specific function^b^ (*n* = 90)	− 0.45 (− 0.76 to − 0.14)	0.36 (− 0.07 to 0.80)	− 0.27 (− 0.96 to 0.41)	0.02 (− 0.56 to 0.60)	− 0.28 (− 0.55 to − 0.01)
Global perceived effect^c^, Odds ratio (*n* = 89)	− 0.45 (− 0.77 to − 0.14)	1.21 (0.57 to 2.57)	0.53 (0.32 to 0.90)	0.67 (0.44 to 1.01)	0.88 (0.66 to 1.16)

*Outcome at 12 months:

^a^Health-related quality of life, EQ5D, range − 0.28 to 1.00,

^b^Patient-specific functional scale, PSFS, range 0–10,

^c^Global perceived effect, GPE, dichotomized: perceived treatment effect or not perceived no treatment effect

## Discussion

The results do not indicate clinically significant mediating effects of pain self-efficacy on the relation between baseline pain intensity and 12 months outcome in this sample of patients seeking physical therapy for musculoskeletal pain. In a subgroup with low baseline self-efficacy, the mediating effect of pain self-efficacy was substantial and clinically significant for patient-specific function. To our knowledge, this is the first prospective study exploring pain self-efficacy as mediator of health-related quality of life, patient-specific function and global perceived effect for patients seeking physical therapy for musculoskeletal pain. We will discuss our results in relation to previous studies that have assessed self-efficacy as a mediator in different pain conditions.

Firstly, the mediating effect of pain self-efficacy on the relation between pain intensity and health-related quality of life was marginal in the present study. Although the results imply a statistically significant mediating effect, the estimates are small and suggest that a minimal important improvement in health-related quality of life [28] would demand an out-of-reach improvement in pain self-efficacy for this patient sample. A minimal important difference for health-related quality of life registered by EQ5D has been reported to be between 0.07 and 0.08 [28]. We calculated the change in self-efficacy needed to produce 0.08 points change in EQ5D and found that an improvement in pain self-efficacy equivalent to 4.2 on the scale (ranging from 0 to 12) would lead to clinically important increases in health-related quality of life. For comparison, the baseline level of the self-efficacy scale was 9.5 (SD 2.6) in this sample (Table 3), implying a ceiling effect, since the average participant could improve maximum 2.5 points. The subgroup analysis indicates a larger mediation effect, but still not likely of clinical importance for health-related quality of life outcomes for patients with low self-efficacy at baseline. In line with the present results, a previous prospective study indicated that although pain self-efficacy mediates health-related quality of life outcomes in headache, effects were small and probably not clinically significant [16]. This may indicate that pain self-efficacy is not a relevant or sufficient treatment target in the present sample. For most patients seeking physical therapy with musculoskeletal symptoms and with characteristics similar to patients in the current sample, pain self-efficacy at 3 months does not seem to be an important mediator for the relation between pain intensity and health-related quality of life at 12 months.

The overall results for patient-specific function were comparable to results for health-related quality of life. The mediating effect of pain self-efficacy on the relation between pain intensity and patient-specific function was statistically significant but small in the full sample (Table 4). However, the magnitude of the mediating effect was substantial in the subgroup with low pain self-efficacy at baseline. Confidence intervals were wide, indicating uncertain estimates probably due to the small sample size in the subgroup. Given that the minimal clinical important difference on the patient-specific functional scale has been suggested to be between 2 and 3 for musculoskeletal conditions [29] and considering the effect estimate and the upper limit of the confidence interval, the mediating effect of pain self-efficacy is likely to have clinical importance in this subgroup of patients. This is in accordance with results from a meta-analysis of mediation studies where pain self-efficacy mediated the effect of pain intensity on disability in patients with low back pain or neck pain [20]. Standardized regression coefficient was 0.23 in the meta-analysis. This effect estimate can be comparable to the estimated mediating effect for the subgroup in the present study. A causal mediation analysis nested in a previous randomized controlled trial found that self-efficacy mediated the effect of education and graded sensorimotor training on disability, − 0.84 [21]. Disability was measured by the Roland-Morris disability questionnaire and the patients included had chronic low back pain. These results support the active role of pain self-efficacy but cannot be directly compared to our results, since the present study had musculoskeletal pain intensity as exposure while the RCT compared low back pain patients randomly assigned to sham treatment with patients receiving an intervention targeting self-efficacy specifically. Moreover, the participants in the low back pain study had relatively lower pain self-efficacy and higher pain intensity at baseline [21] compared to participants in the present study and consequently a better potential for improvement.

Our results indicated no mediating effect of pain self-efficacy on the relation between pain intensity and global perceived effect. To our knowledge, this has not been examined in previous studies. In summary, the individualized outcome was mediated by pain self-efficacy in a subgroup, and there were no substantial mediating effects for the generic outcomes.

### Strengths and limitations

The temporal order of exposure, mediator and outcome is an important strength and enhances the causal interpretability of this mediation analysis. The sequential order with registrations of exposure and confounders at baseline, mediator at 3 months and the outcome at 12 months entail temporal precedence, which is an important prerequisite for causal interpretation [18, 33].

As this study is observational, confounding presents a challenge; however, we adjusted for a range of factors following careful consideration. On the other hand, observational designs are more naturalistic than experiments as they are integrated into the daily practice where the actual primary care patients are studied. These are the patients that clinicians meet in their practice. However, the patients in the present sample did not report severe pain or reduced health-related quality of life at baseline, causing a limited potential for improvement. The sample from this study was comparable to the FYSIOPRIM population with musculoskeletal pain and complete baseline data (Online resource E). Since there were no differences in baseline characteristics between the included sample and the total population, the risk of selection bias is considered low. Both the mediator and the outcomes changed most between baseline and 3 months (Table 3) and we do not know if the mediator changed before the outcomes, which is a prerequisite for our causal model. More frequent measurements could have described the sequential ordering between the mediator and the outcome. Further, the four-way decomposition model allows for causal interpretation only given that the assumptions are fulfilled [34]. Though we have applied DAGS and adjusted for potential known confounders, there may be residual confounding, i.e. due to misclassification, imperfect measurements, over- or under-adjustment in models, or unknown confounders [42]. There is a risk for overadjustment in the current study, but we considered the advantages of including the relatively extensive set of confounders to surpass this risk. However, the sensitivity analyses supported the main analysis, since adding confounders or dichotomizing variables did not change the results.

The relation between pain intensity and health-related quality of life is complex [7], and in the present study, the direct effect of pain intensity on the outcomes was marginal, which may have weakened the studies’ ability to explore mediating effects. Another challenge was the small change in pain self-efficacy through the treatment period, probably influenced by the ceiling effect since mean mediator score was relatively high at inclusion. This may imply that pain self-efficacy was a potential mediator for only a small proportion of the sample. The small size of the subgroup implies cautious interpretation of the results from subgroup analysis. We do also not know to what extent the physical therapy treatment included cognitive behavioral treatment elements targeting pain self-efficacy directly.

The use of instruments with good methodological quality for outcome evaluation strengthens the study. However, measuring pain intensity by numeric rating scale [43] and pain self-efficacy by 2-items PSEQ [44] may have some shortcomings that influenced the results. On the other hand, in the total sample, the results were similar for all outcomes and across sensitivity analyses, which strengthens the conclusion.

### Implications

The overall results do not support that pain self-efficacy mediates the influence of pain intensity on outcome after 12 months in this sample of patients seeking physical therapy, with low to moderate intensity musculoskeletal pain and without substantially reduced pain self-efficacy at baseline. The subgroup analyses indicated that an increase in pain self-efficacy when pain self-efficacy is substantially reduced, is one mechanism that may improve patient-specific function after physical therapy.

Larger studies with more frequent measurement points are warranted to follow the change processes during treatment. Additionally, the role of potential mediators should be explored in samples with more explicit improvement potential than the current sample, which mainly included patients with moderate to high pain self-efficacy.

## Conclusion

The mediating effect of pain self-efficacy on the relation between pain intensity at baseline and outcome at 12 months was below limits for clinical significance in this sample of patients seeking physical therapy for musculoskeletal pain. In a subgroup with reduced pain self-efficacy at baseline, the indirect effect of pain self-efficacy on the relation between pain intensity and patient-specific function was significant, implying that in patients with low scores of pain self-efficacy, this may be a factor worth addressing.

## Supplementary Information

Below is the link to the electronic supplementary material.

Supplementary Material 1

## Data Availability

The dataset analyzed in this study are not publicly available due to permission has not been applied for from neither the participants nor the Ethical Committee. The code for analysis is available on reasonable request from the corresponding author.

## Declarations

### Reporting standard

The study was conducted and reported according to the AGReMA standard (Cashin et al. 2022 #2068), and the completed checklist is attached as a supplement (Online resource C).
