# Supplementary material for: Can pain self-efficacy mediate the influence of pain intensity on outcomes for patients with musculoskeletal pain? A prospective mediation analysis with 12 months follow-up
Source: Qual Life Res. 2026 Jan 3;35(1):21. doi: 10.1007/s11136-025-04118-3 (PMC12764639; doi:10.1007/s11136-025-04118-3)
Supplement: Supplementary file 1 — Supplementary Material 1 [file 11136_2025_4118_MOESM1_ESM.docx]

| **Online resource A.** Sensitivity analyses for health-related quality of life and function with pain-self efficacy at 3 months dichotomized, as due to non-normal distribution of the mediator variable pain self-efficacy. | | | | | |
| --- | --- | --- | --- | --- | --- |
| Mediating effect of pain self-efficacy at 3 months on outcome | Exposure* (pain intensity)-mediator** effect (path A) | Mediator**-outcome effect  (path B) | Average total effect | Average direct effect | **Pure indirect effect** |
| Health-related quality of life^1^  (n=422), β (95% CI), if otherwise not stated | OR=0.85  (0.71 to 1.03) | 0,03  (-0,1 to 0,15) | -0,01  (-0,03 to 0,01) | 0,15  (-0,22 to 0,52) | -0,001  (-0,002 to -0,001) |
| Patient-specific function^2^  (n = 418) , β (95% CI) | Not applicable (as above) | 0.38  (-1.68 to 2,44) | 0.02  (-0.24 to 0.29) | 1,11  (-4.99 to 7.22) | -0.01  (-0.03 to 0.01) |
| *Exposure: Pain intensity, Numeric pain rating scale, range 0-10. Lower score = better.  **Mediator: Pain self-efficacy, 2-item pain self-efficacy questionnaire, 0-12. Higher score = better. Dichotomized as high (8-12) versus low (0-7) self-efficacy  1) Health-related quality of life at 12 months, EQ5D, range -0.28 to 1.00). Higher score = better. Β-estimate.  2) Patient-specific functional scale at 12 months, PSFS, range 0-10. Higher score = better. Β-estimate.  Β = coefficient from the regression analyses | | | | | |

| **Online resource B** Sensitivity analyses for health-related quality of life and function with the outcome variables dichotomized at 12 months, due to non-normal distribution of the outcome variables. | | | | | |
| --- | --- | --- | --- | --- | --- |
| Mediating effect of pain self-efficacy at 3 months on outcome | Exposure* (pain intensity)-mediator** effect (path A) | Mediator**-outcome effect  (path B) | Average total effect | Average direct effect | **Pure indirect effect** |
| Health-related quality of life^1^  (n=422) , | β =-0.16  (-0,27 to -0,05) | 1.44  (1.08 to 1.93) | 0.91  (0.71 to 1.18) | 1.06  (0.86 to 1.31)) | 0.94  (0.89 to 0.996) |
| Patient-specific function^2^  (n = 418) , OR(95% CI) | Not applicable (as above) | 1.43  (0.90 to 2.28) | 0.99  (0.75 to 1.30) | 1.13  (0.92 to 1.38) | 0.92 (0.86 to 0.98) |
| *Exposure: Pain intensity, Numeric pain rating scale, range 0-10. Lower score = better.  **Mediator: Pain self-efficacy, 2-item pain self-efficacy questionnaire, 0-12. Higher score = better.  1) Health-related quality of life, EQ5D, range -0.28 to 1.00). Dichotomized as low (EQ5D=-0.28 to 0.77) and high (EQ5D=0.78 to 1.00) health-related quality of life  2) Patient-specific functional scale, PSFS, range 0-10. Dichotomized as poor (PSFS=0-7) and good (PSFS=8-10) function.  β= coefficient from the regression analyses  OR= odds ratio | | | | | |


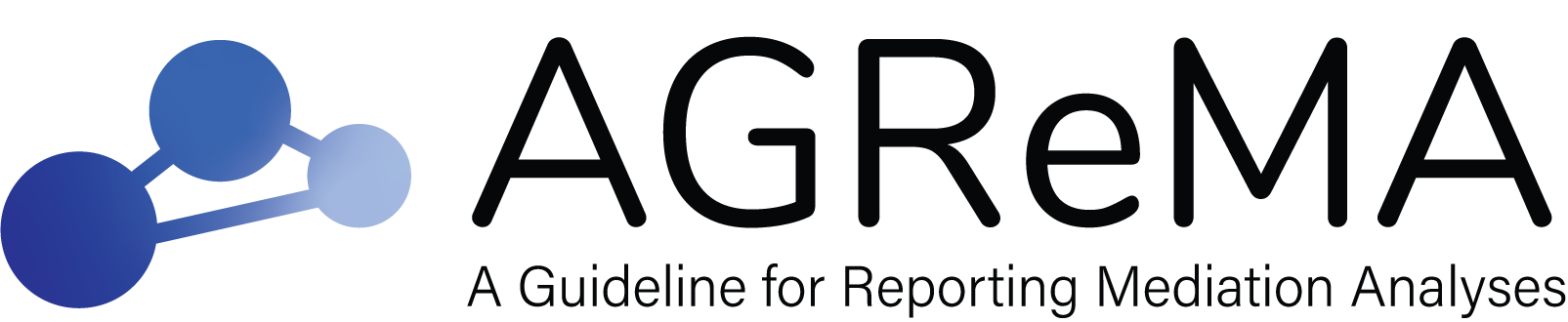
 **Online resource C**

| **Section/Topic** | **Item Number** | **Item Description** | **Reported on page No** |
| --- | --- | --- | --- |
| **Title and abstract** | | | |
| Title | 1 | Identify that the study uses mediation analysis | 1 |
| Abstract | 2 | Provide a structured summary of the objectives, methods, results, and conclusions specific to mediation analyses | 1 |
| **Introduction** | | | |
| Background and rationale | 3 | Describe the study background and theoretical rationale for investigating the mechanisms of interest. Include supporting evidence or theoretical rationale for why the intervention or exposure might have a causal relationship with the proposed mediators. Include supporting evidence or theoretical rationale for why the mediators might have a causal relationship with the outcomes | 2-3 |
| Objectives | 4 | State the objectives of the study specific to the mechanisms of interest. The objectives should specify whether the study aims to test or estimate the mechanistic effects | 3 |
| **Methods** | | | |
| Study registration | 5 | If applicable, provide references to any protocols or study registrations specific to the mediation analysis, and highlight any deviations from the planned protocol | Title page, and page 4 |
| Study design and source of data | 6 | Specify the design of the original study that was used in mediation analyses and where the details can be accessed, supported by a reference. If applicable, describe study design features that are relevant to mediation analyses | 4 |
| Participants | 7 | Describe the target population, eligibility criteria specific to mediation analyses, study locations, and study dates (start of participant enrolment and end of follow-up) | 4 |
| Sample Size | 8 | State whether a sample size calculation was conducted for mediation analyses. If so, explain how it was calculated | 4 |
| Effects of interest | 9 | Specify the effects of interest | 5-6 |
| Assumed causal model | 10 | Include a graphic representation of the assumed causal model including the exposure, mediator, outcome, and possible confounders | 5 |
| Causal assumptions | 11 | Specify assumptions about the causal model | 8 |
| Measurement | 12 | Clearly describe the interventions or exposures, mediators, outcomes, confounders, and moderators that were used in the analyses. Specify how and when they were measured, the measurement properties, and whether blinded assessment was used | 5-7, Table 1 |
| Measurement levels | 13 | If relevant, describe the levels at which the exposure, mediator, and outcome were measured | Tables 1 and 3-5 |
| Statistical methods | 14 | Describe the statistical methods used to estimate the causal relationships of interest. This description should specify analytical strategies used to reduce confounding, model building procedures, justification for the inclusion or exclusion of possible interaction terms, modelling assumptions, and methods used to handle missing data. Provide a reference to the statistical software and package used | 7-9 |
| Sensitivity analyses | 15 | Describe any sensitivity analyses that were used to explore causal or statistical assumptions and the influence of missing data | 9 |
| Ethical approval | 16 | Name the institutional research board or ethics committee that approved the study. Provide a description of participant informed consent or ethics committee waiver of informed consent | 20 |
| **Results** | | | |
| Participants | 17 | Describe baseline characteristics of participants included in mediation analyses. Report the total sample size and number of participants lost during follow-up or with missing data | 4, 10  Tables 4-5. |
| Outcomes and estimates | 18 | Report point estimates and uncertainty estimates for the exposure-mediator and mediator-outcome relationships. If inference concerning the causal relationship of interest is considered feasible given the causal assumptions, report the point estimate and uncertainty estimate | 11-12    Tables 4-5. |
| Sensitivity parameters | 19 | Report the results from any sensitivity analyses used to assess robustness of the causal or statistical assumptions, and the influence of missing data | 11 |
| **Discussion** | | | |
| Limitations | 20 | Discuss the limitations of the study including potential sources of bias | 15-16 |
| Interpretation | 21 | Interpret the estimated effects considering the study’s magnitude and uncertainty, plausibility of the causal assumptions, limitations, generalizability of the findings, and results from relevant studies | 14-16 |
| Implications | 22 | Discuss the implications of the overall results for clinical practice, policy, and science | 16 |
| **Other information** | | | |
| Funding and role of  sponsor | 23 | List all sources of funding or sponsorship for the mediation analysis and the role of the funders/sponsors in the conduct of the study, writing of the manuscript, and decision to submit for publication. | 20 |
| Conflicts of interest and financial disclosures | 24 | State any conflicts of interest and financial disclosures for all authors | 20 |
| Data and code | 25 | Authors are encouraged to provide a statement for sharing data and code for the mediation analysis | 20 |

*From:*  Lee H, Cashin AG, Lamb SE, Hopewell S, Vansteelandt S, VanderWeele TJ, et al. A Guideline for Reporting Mediation Analyses of Randomized Trials and Observational Studies. The AGReMA Statement. JAMA. 2021;326(11):1045–1056. doi:10.1001/jama.2021.14075

AGReMA is designed for articles that report mediation analyses of randomized trials or observational studies.

For more information, visit: [agrema-statement.org](https://agrema-statement.org/)

**Online resource D: Short narrative of literature supporting the selection of confounders.**

Confounders were selected supported by previous studies and reasoning connected to clinical course and prognosis in musculoskeletal pain. The following factors were identified as significant for prognosis in systematic reviews: Symptom duration [2-4], age [2, 3], psychological distress [3, 4], and number of pain sites [2, 3, 5]. Gender [6], comorbidity [7], BMI [8], educational level [9], pain medication [10], pain self-efficacy [11], fear of movement [4], physical activity level [12], and sleep [10] was also identified as potential confounders based on previous studies. We reserved two potential confounders for sensitivity analysis; sleep and number of pain sites, to reduce the risk of collinearity and to estimate the influence of residual confounding.

[1] A. G. Cashin, J. H. McAuley, and H. Lee, "A reporting guideline for randomized trials and observational studies using mediation analysis: AGReMA," *Nat. Med.,* vol. 28, no. 3, pp. 432-434, Mar 2022, doi: 10.1038/s41591-021-01674-2.

[2] G. H. Valentin *et al.*, "Prognostic factors for disability and sick leave in patients with subacute non-malignant pain: a systematic review of cohort studies," *BMJ Open,* vol. 6, no. 1, p. e007616, Jan 6 2016, doi: 10.1136/bmjopen-2015-007616.

[3] D. J. Green *et al.*, "Clinical course and prognostic factors across different musculoskeletal pain sites: A secondary analysis of individual patient data from randomised clinical trials," *Eur. J. Pain,* vol. 22, no. 6, pp. 1057-1070, Jul 2018, doi: 10.1002/ejp.1190.

[4] N. B. de Vos Andersen, P. Kent, J. Hjort, and D. H. Christiansen, "Clinical course and prognosis of musculoskeletal pain in patients referred for physiotherapy: does pain site matter?," *BMC Musculoskelet. Disord.,* vol. 18, no. 1, p. 130, Mar 29 2017, doi: 10.1186/s12891-017-1487-3.

[5] M. Artus, P. Campbell, C. D. Mallen, K. M. Dunn, and D. A. van der Windt, "Generic prognostic factors for musculoskeletal pain in primary care: a systematic review," (in eng), *BMJ Open,* vol. 7, no. 1, p. e012901, Jan 17 2017, doi: 10.1136/bmjopen-2016-012901.

[6] C. K. Peterson, B. K. Humphreys, J. Hodler, and C. W. Pfirrmann, "Gender differences in pain levels before and after treatment: a prospective outcomes study on 3,900 Swiss patients with musculoskeletal complaints," *BMC Musculoskelet. Disord.,* vol. 13, p. 241, Dec 5 2012, doi: 10.1186/1471-2474-13-241.

[7] A. L. Nordstoga, T. I. L. Nilsen, O. Vasseljen, M. Unsgaard-Tondel, and P. J. Mork, "The influence of multisite pain and psychological comorbidity on prognosis of chronic low back pain: longitudinal data from the Norwegian HUNT Study," *BMJ Open,* vol. 7, no. 5, p. e015312, Jun 6 2017, doi: 10.1136/bmjopen-2016-015312.

[8] F. Pan *et al.*, "Associations Between Fat Mass and Multisite Pain: A Five-Year Longitudinal Study," *Arthritis Care Res. (Hoboken),* vol. 69, no. 4, pp. 509-516, Apr 2017, doi: 10.1002/acr.22963.

[9] C. E. Dionne, M. Von Korff, T. D. Koepsell, R. A. Deyo, W. E. Barlow, and H. Checkoway, "Formal education and back pain: a review," *J. Epidemiol. Community Health,* vol. 55, no. 7, pp. 455-68, Jul 2001, doi: 10.1136/jech.55.7.455.

[10] N. Pourbordbari, M. B. Jensen, J. L. Olesen, S. Holden, and M. S. Rathleff, "Prognosis and bio-psycho-social prognostic factors in children and adolescents with musculoskeletal pain consulting general practice," *Eur. J. Pediatr.,* vol. 184, no. 6, p. 384, Jun 2 2025, doi: 10.1007/s00431-025-06217-2.

[11] J. Martinez-Calderon, C. Zamora-Campos, S. Navarro-Ledesma, and A. Luque-Suarez, "The Role of Self-Efficacy on the Prognosis of Chronic Musculoskeletal Pain: A Systematic Review," *J. Pain,* vol. 19, no. 1, pp. 10-34, Jan 2018, doi: 10.1016/j.jpain.2017.08.008.

[12] L. Palmlof, L. W. Holm, L. Alfredsson, C. Magnusson, E. Vingard, and E. Skillgate, "The impact of work related physical activity and leisure physical activity on the risk and prognosis of neck pain - a population based cohort study on workers," *BMC Musculoskelet. Disord.,* vol. 17, p. 219, May 20 2016, doi: 10.1186/s12891-016-1080-1.

**Online resource E**

|  | **Sample 1 for analysis**  **(n = 422)** | **Sample 2 with musculoskeletal pain (n=1062)** |
| --- | --- | --- |
| **Age, mean (SD)** | **48.6 (16.6)** | **45.2 (16.5)** |
| **Female gender, n (%)** | **302 (71.6)** | **721 (67.9)** |
| **Body mass index, mean (SD)** | **26.2 (4.7)** | **26.1 (4.8)** |
| **Higher education, n (%)** | **259 (61.4)** | **635 (59.8)** |
| **Physically inactive, n (%)^a^** | **101 (23.9)** | **265 (25.0)** |
| **Fear of movement, mean (SD)^b^ (0-10)** | **3.1 (2.8)** | **3.3 (2.8)** |
| **Use of pain medication last week, n (%)** | **190 (45.0)** | **449 (42.3)** |
| **Number of pain sites (0-10) , mean (SD)** | **3.0 (2.3)** | **3.0 (2.2)** |
| **Pain duration, n (%)** |  |  |
| **< 3 months** | **34 (8.1)** | **89 (8.4)** |
| **3-12 months** | **180 (42.7)** | **455 (42.8)** |
| **> 12 months** | **208 (49.3)** | **518 (48.8)** |
| **Mental distress, HSCL (1.0-4.0)^c^, mean (SD)** | **1.65 (0.54)** | **1.65 (0.53)** |
| **Sleep problems, n (%) ^d^** | **150 (35.6)** | **358 (33.7)** |
| **Pain intensity (0-10)^e^** | **4.3 (2.2)** | **4.3 (2.2)** |
| **Pain self-efficacy (0-12)^f^** | **9.5 (2.6)** | **9.4 (2.7)** |
| **Health related quality of life, (-0.285-1.00)^g^** | **0.65 (0.18)** | **0.66 (0.18)** |
| **Patient Specific Functional Scale, range (0-10)^h^** | **3.7 (2.4)** | **3.8 (2.4)** |

**Online resource F**

| **Table 4. Effect decomposition in whole sample: Mean difference on outcome health-related quality of life, patient-specific function and global perceived effect. Sensitivity analyses with the confounders sleep and number of pain sites.** | | | | | |
| --- | --- | --- | --- | --- | --- |
| **Mediating effect of pain self-efficacy at 3 months on outcome** | **Reference interaction** | **Mediated interaction** | **Average total effect** | **Average direct effect** | **Pure indirect effect** |
| **Health-related quality of life^c^**  **(n=422)**  **β (95% CI)** | **0.001 (-0.004 to 0.005)** | **-0.0003 (-0.002 to 0.001)** | **-0.013**  **(-0.03 to 0.00)** | **-0.01**  **(-0.02 to 0.01)** | **-0.01**  **(-0.01 to 0.00)** |
| **Patient-specific function^d^**  **(n = 418)**  **β (95% CI)** | **-0.02 (-0.10 to 0.05)** | **0.01 (-0.02 to 0.04)** | **-0.01**  **(-0.27 to 0.25)** | **0.09**  **(-0.17 to 0.34)** | **-0.08**  **(-0.15 to -0.01)** |
| **Global perceived effect^e^**  **(n = 416)**  **Odds ratio** | **0.98 (0.88 to 1.08)** | **1.01 (0.98 to 1.04 )** | **0.84**  **(0.68 to 1.05)** | **0.89**  **(0.73 to 1.08)** | **0.97**  **(0.92 to 1.01)** |
| 1. **Exposure: Pain intensity, Numeric pain rating scale, range 0-10. Lower score = better.** 2. **Mediator: Pain self-efficacy, 2-item pain self-efficacy questionnaire, 0-12. Higher score = better.** 3. **Health-related quality of life, EQ5D, range -0.28 to 1.00). Higher score = better. β-estimate.** 4. **Patient-specific functional scale, PSFS, range 0-10. Higher score = better. β-estimate.** 5. **Global perceived effect, GPE, dichotomized: perceived treatment effect or not. OR=odds ratio** | | | | | |
